# Description of Limit States in the Subsurface Layer of Loosened Subsoil in View of Critical State Soil Mechanics

**DOI:** 10.3390/ma14237288

**Published:** 2021-11-28

**Authors:** Jan Fedorowicz, Lidia Fedorowicz, Marta Kadela

**Affiliations:** 1 Faculty of Architecture, Civil Engineering and Applied Arts, University of Technology, ul. Rolna 43, 40-555 Katowice, Poland; jankfedorowicz@gmail.com (J.F.); lidiafedorowicz@gmail.com (L.F.); 2 Building Research Institute (ITB), ul. Filtrowa 1, 00-611 Warsaw, Poland

**Keywords:** mining area, building-mining subsoil system, numerical analysis, finite element model

## Abstract

The article aims to present an effective numerical method for the behaviour analysis and safety assessment of a subsurface layer of subsoil in the existing or predicted states of mining and post-mining deformations. Based on our own analytical record, using the equations of the Modified Cam-Clay model, the description of limit states in the subsurface layer of subsoil was validated, making it consistent with in situ observations. The said effect was demonstrated by comparing numerical analyses of the subsoil layer subjected to the limit state, using the Modified Cam-Clay (MCC) model and the Coulomb-Mohr model (C-M). The article also presents the applicability potential of the numerical analysis of the loosened subsoil layer for the assessment of protection elements (e.g., geo-matresses) used under linear structures in the areas subjected to mining and post-mining impacts.

## 1. Introduction

**Figure 1:**
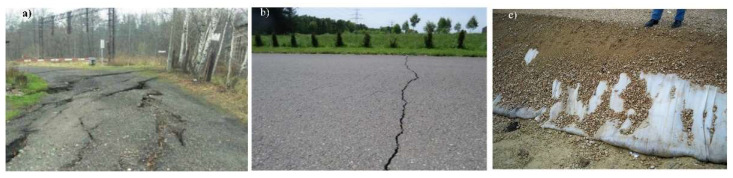
(**a**) The effect of the large mining displacement on the surface area [18], (**b**) the effects of mining impact on the road pavement (own photo), (**c**) protection of the A1 motorway in the mining area (own photo).

In Poland, in 2020, hard coal mining amounted to approximately 54.4 million tons, and in the previous year, over 61.6 million tons of hard coal. Most of the exploitation is carried out under urbanised areas [1]. The rock mass displacement or from drainage processes carried out in a mine affects the continuous or discontinuous deformations of the surface of the mining area [2,3]. The issue of mining impacts on the subsoil and building structures were described in detail in [4,5]. Examples of damage in buildings were presented, i.e., by Can et al. [6], Hu et al. [3], Liu et al. [7] and Lipecki et al. [8], while in the bridge structures, i.e., by Bętkowski [9] and Parkasiewicz et al. [10]. The exploitation of hard coal can cause large-scale ground displacement. Figure 1a presents local effect exceeding the permissible values of horizontal strains (*ε_x_*) due to soil loosening. This is related to reaching the limit state, which was presented in detail in [11,12,13,14]. The critical state models were presented by Barnes [15], Braja [16] and Whitlow [17].

Adhelsohn et al. [19], Kotyrba and Kowalski [20], and Grygierek and Zięba [21] reported on the mining impact on the road pavements. Adhelsohn et al. [19] analysed factors, which have most impacted the highway alignments. While Kotyrba and Kowalski [20] and Grygierek and Zięba [21] analyzed the damage (fissures and stages) on the A4 motorway and the adjacent parking lot in the mining area due to the geological and mining conditions of the deformation and the collected observational and measurement data. Figure 1b presents the cracks across the road due to mining impact. Therefore, for many cases, it is necessary to use protection, e.g., geosynthetics [22] or geo-mattresses [23]—Figure 1c—the cost of which is considerable [24].

Nowadays, numerical analyses play an increasingly important role in designing and assessing the construction structure and phenomena occurring in subsoil [25,26,27]. For example, Shu et al. [25] used different constitutive models to recreate undisturbed subsoils’ stress–strain strength characteristics. Kadela and Fedorowicz [26,27] and Kadela [28] used the MCC model to recreate the subsoil behaviour of the road pavement-subsoil system under the real load from the vehicle wheels. Nascimbene [29] attempted to recreate the influence of seismic earthquakes on the building. The results of the simulation of underground mining subsidence and its induced damages on buildings were reported, i.e., by Álvarez-Fernández [30], Cai [31] and Saeidi [32]. The spread of deformation state in the rock mass caused by mining exploitation brings about changes in the strain state in the subsoil. It also causes changes in the initial stress state. An adequate assessment of these states depends on the used constitutive model that simulates soil's real behaviour. Kwiatek et al. [33] and Ju and Xu [34] tried to recreate the subsoil and the surface area behaviour during the mining exploitation in laboratory tests. The characteristic phenomena observed for mining subsoil there were recreated in laboratory tests. Hejmanowski et al. [35,36] investigated and recreated building damage risk on mining areas by GIS application. Numerical, experimental forecasting of surface deformation due to the exploited rock mass was investigated by Kwaśniewski and Wang [37,38]. Fedorowicz [39] used the boundary conditions changing in time (as the exploitation progresses) to solve the boundary problem which is the mining subsoil in the numerical model. The assumption of dividing the predicted subsoil mining surface deformations into vertical and horizontal (assumed as horizontal strains) displacements is consistent with the simplified boundary conditions described, among others, by Kwiatek [4]. Using the non-simplified boundary conditions of the mining subsoil, Florkowska [40] used an elastic and elastic-plastic constitutive model to assess the behavior of mining subsoil in a 3D state. However, as a result of applying the commonly used model (C-M), the limit state obtained in the subsurface layers is often inconsistent with the behaviour of the subsoil in the in-situ state (see Figure 1a,b). So, the results of numerical (C-M) analyses often indicate the need to use (mainly for road pavements in mining and post-mining areas) complex and costly protections (Figure 1c).

Therefore, this study aimed develop a method that allows an adequate assessment of the effects of mining and post-mining deformations that occur in the subsoil subjected to mining impact. In this purpose, numerical analysis was carried out using the commonly used Coulomb-Mohr model and the Modified Cam-Clay (MCC) critical state model (so far not used in practice in this type of analysis). The MCC model was presented in detail in [16,41,42] and currently is re-analysed frequently by researchers. For example, Silverini and Abou-Samra [43] used the Modified Cam Clay model (MCC) to assess the consolidation state of the subsoil in expansive situations. Gaone et al. [44] determined MCC model parameters in back-analyses using the self-boring pressure meter test data. Also, an attempt to use the MCC model under cyclic load was presented by Goldstein et al. [45]. Moreover, MCC model was used describe of behavior of granular materials under static monotonic [46,47] and cyclic loadings [48,49]. Ilyashenko and Kuznetsov [50] reviewed mathematical models of granular materials using the critical state concept.

In this study, the behaviour of the mining subsoil was analysed (using the FEM model) in order to adequately assess the behavior of subsurface layers and the safety of structures subject to local mining deformations. The assessment of the adequacy of the computational model was combined with the calibration requirement—i.e., to reproduce the phenomena known from in situ observations and laboratory tests, using a selected constitutive model. This study used a description of the states existing in the mining subsoil confirmed by laboratory tests [33,43]. As the result of the conducted analysis, a method of realistic assessment for the mining areas' safety was based on the MCC model.

## 2. Numerical Model

### 2.1. Description of Creating a Computational Model

**Figure 2:**
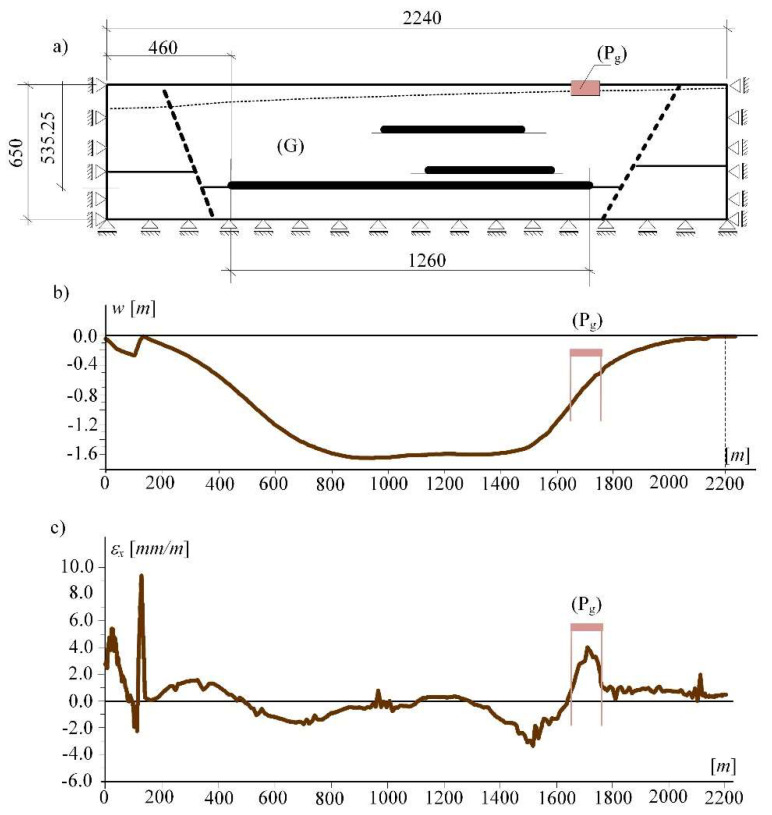
(**a**) Model of rock mass (G) and the studied mining subsoil (P_g_), (**b**) state of the surface above the exploitation area called subsidence, (**c**) horizontal strains.

Figure 2a presents the rock mass (G) with specific geological and mining conditions and the area of the so-called mining subsoil (P_g_) that transmits the mining deformations to the building structure. The steady-states (see Figure 2b,c) result from an incremental procedure (using the Hook-Brown model) that simulates changes in the state of stress and strains (and changes in deformation of the land surface) occurring during exploitation.

**Figure 3:**
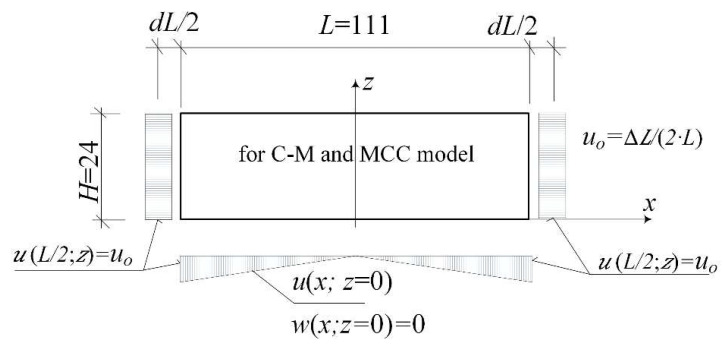
Loosening of the mining subsoil (P_g_) in the (2D) description.

For the studied area (P_g_) the kinematic boundary conditions realising the states of loosening or compaction of soil were introduced. The kinematic boundary conditions were used according to Fedorowicz [39]. It is consistent with the literature [4,5] and based on earlier authors experiences in numerical simulations [39,51]). Figure 3 presents the (P_g_)-area in the (2D) state, its size and boundary conditions in a numerical model of the loosening subsoil. The size of the subsoil area was assumed according to Fedorowicz [51].

Two different models—elastic-plastic model C-M (case I) and critical state model MCC (case II)—were considered to describe of the behaviour of soil. The assumed parameters of constitutive models are shown in Table 1 and Table 2, respectively. All numerical calculations were performed using the Abaqus FEM software [52].

### 2.2. Description of the C-M and MCC Models

#### 2.2.1. C-M Model

**Figure 4:**
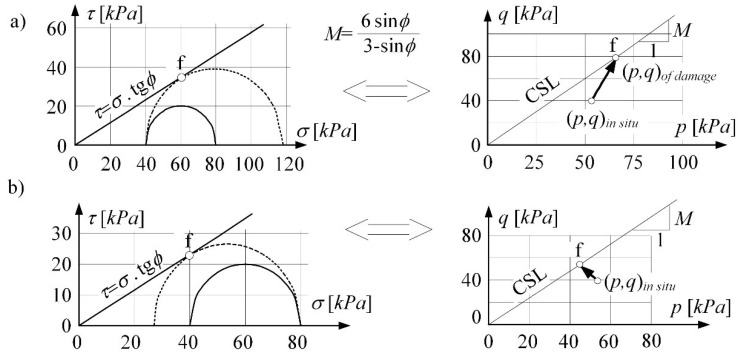
Damage in triaxial tests: (**a**) in shear with drainage, (**b**) when horizontal stress decrease.

In case I was considered elastic-plastic model (C-M), see Figure 4. The Coulomb-Mohr criterion is the basic strength criterion. The damage obtained in triaxial tests when the horizontal stress decrease is shown in Figure 4b. This is equivalent to the state of soil loosening in the mining (or post-mining) subsoil. 

**Figure 5:**
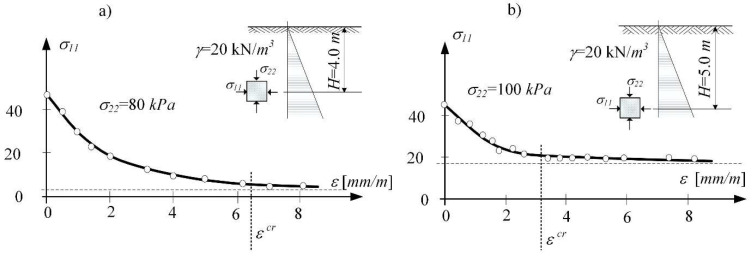
Stress-strain diagram of soil samples (clayed sand) subjected to loosening obtained in (2D) analysis for different depths: (**a**) H = 4.0 m, (**b**) H = 5.0 m [39].

The stress path is mapping the loosening process (the compaction process) taking place in the subsoil layer. In the (*p*, *q*) invariants system, the stress path turns to the left and moves towards the critical state line (CSL) at the boundary surface. The stress-strain curve of the soil loosening was obtained by Kwiatek et al. [33,53]. Figure 5 presents the reconstruction of this curve in (2D) numerical analysis [39]. It shows the formation sequence of the limit state in the real consolidated subsoil [16,41]. Moreover, it allows identifying (for the selected depth *z*) the strain initiating the process of critical loosening of soil *ε^cr^*. The horizontal strain associated with the flow (*ε^cr^*) is different depending on the ratio of horizontal to vertical stress in the in situ state. That means the state depends on the consolidation rate of the samples (e.g., different over-consolidation ratio OCR).

The Coulomb-Mohr model is described by the function of stress invariants (*p*, *q*) and the function of hardening parameter *κ*^(*i*)^ according to Equation (1):

(1) $$f\left(\sigma ;\kappa^{\left(i\right)}\right)=f\left(p,q;\kappa^{\left(i\right)}\right)$$

The associated flow rule and isotropic hardening, expressed as a function of plastic volumetric strain characterize the model. The model has been described by a system of Equations (2) and (3), consisting of the laws of strain additivity, elasticity and flow, compliance conditions, and hardening functions.

(2) $$\delta \varepsilon =\delta \varepsilon^{e}+\delta \varepsilon^{p},\;\delta \sigma =D^{e}\cdot \delta \varepsilon^{e},\delta \varepsilon^{p}=a_{f}\cdot \delta \lambda ,\;df=a_{f}\cdot \delta \sigma +\frac{\partial f}{\partial \kappa^{\left(i\right)}}=0$$

(3) $$\delta \kappa^{\left(i\right)}={\left\{f\left(\varepsilon^{p}\right)\right\}}^{T}\;\cdot \delta \varepsilon^{p},$$

where:

$$a_{f}=\left\{\frac{\partial f}{\partial \sigma }\right\}={\left\{\frac{\partial f}{\partial p}\cdot \frac{\partial p}{\partial \sigma }+\frac{\partial f}{\partial q}\cdot \frac{\partial q}{\partial \sigma }\right\}}^{T}$$

From this system, the incremental constitutive relations for the material were determined by Equation (4).

(4) $$\delta \sigma =D^{e}\cdot \left(\delta \varepsilon -\delta \varepsilon^{p}\right)=D^{ep}\cdot \delta \varepsilon =\left(D^{e}-D^{p}\right)\cdot \delta \varepsilon =\left(D^{e}-\frac{D^{e}\cdot a_{f}\cdot a_{f}^{T}\cdot D^{e}}{a_{f}^{T}\cdot D^{e}\cdot a_{f}+K_{f}}\right)\cdot \delta \varepsilon$$

where: *D^ep^*—is an elastic-plastic matrix and *D^e^*, *D^p^*—elastic and plastic part, respectively, with the description dependent on the constitutive model of the soil; there are for the isotropy of the material and the plane state of strain according to Equation (5) [39,51].

(5) $$D^{e}=\frac{1}{3}\left[\begin{array}{ccc} 3K_{et}+4G_{et} & 3K_{et}-2G_{et} & 0\\ 3K_{et}-2G_{et} & 3K_{et}+4G_{et} & 0\\ 0 & 0 & 3G_{et}\\ 3K_{et}-2G_{et} & 3K_{et}-2G_{et} & 0 \end{array}\right],\;D^{p}=-\frac{1}{H}\left[\begin{array}{ccc} H_{11}^{2} & H_{11}\cdot H_{22} & H_{11}\cdot H_{12}\\ H_{22}\cdot H_{11} & H_{22}^{2} & H_{22}\cdot H_{12}\\ H_{12}\cdot H_{11} & H_{12}\cdot H_{22} & H_{12}^{2}\\ H_{33}\cdot H_{11} & H_{33}\cdot H_{22} & H_{33}\cdot H_{12} \end{array}\right],$$

where *K_et_* and *G_et_*—compression tangent modulus and tangent shear modulus, as a function of mean stress *p* according to Gryczmański [54] and *K_f_*, *H* and *H_ij_*—functions dependent on the assumed constitutive model of soil.

#### 2.2.2. A Layer of Loosened Subsoil

**Figure 6:**
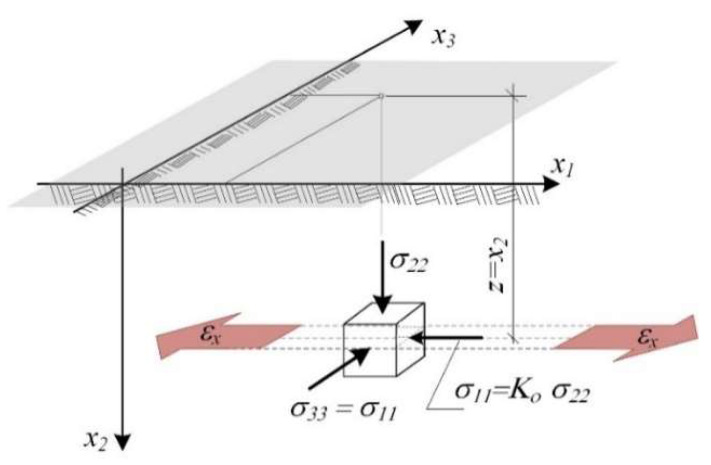
Horizontal loosening of the subsoil layer [39].

In the subsoil layer not loaded with a construction structure, in situ state of stress, caused by self-weight (Figure 6) can be written according to Equation (6).

(6) $$\sigma_{insitu}={\left\{\begin{array}{cccccc} K_{o}\cdot \gamma \cdot z & \gamma \cdot z & K_{o}\cdot \gamma \cdot z & 0 & 0 & 0 \end{array}\right\}}^{T},$$

where *γ*—volumetric weight of soil, *z* = *x*_2_—location of the investigated point and *K_o_*—coefficient of lateral earth pressure at rest.

The main hazard to structures in areas subject to mining impact is related to the soil loosening (or compaction), expressed by horizontal strain *ε_x_* [53]. 

The strain along the *x*_1_ axis was assumed to be *ε*_11_ = *ε_x_* in the investigated subsoil layer (P_g_), where the extreme predicted strain *ε_x_* results from the exploitation process of the rock mass (Figure 6). Strain along the *x*_3_ axis, in the plain strain state, is equal to 0.

The strain occurring in soil *ε_x_* changes the state of stress in the subsoil layer (P_g_). The procedure for determining the stress path in a layer was carried out according to Table 3. Assuming the elastic-plastic model, the change in stress state *δσ^i+^*^1^ caused by the (*i* + 1) increment of strain $\delta \varepsilon_{x}^{i+1}$ can be written in accordance with Equation (7).

(7) $$\delta \sigma^{i+1}={\left\{\begin{array}{cccc} \delta \sigma_{11}^{i+1} & 0 & 0 & \delta \sigma_{33}^{i+1} \end{array}\right\}}^{T}\;\mathrm{where}\;\delta \varepsilon^{i+1}={\left\{\begin{array}{ccc} \delta \varepsilon_{x}^{i+1} & \delta \varepsilon_{22}^{i+1} & 0 \end{array}\right\}}^{T}$$

The changes in the stress state, which form a path of stress, can be presented (taking into account the formulas (2)–(5)) in the form of Equation (8).

(8) $$\delta \sigma^{i+1}=D^{ep}\cdot \delta \varepsilon^{i+1}=\left(D^{e}-D^{p}\right)\cdot \delta \varepsilon^{i+1}\;=f_{1}\left(\sigma^{i};{\left(H_{ij},H,K_{et},G_{et}\right)}^{i}\right)\cdot \delta \varepsilon_{22}^{i+1}+f_{2}\left(\sigma^{i};{\left(H_{ij},H,K_{et},G_{et}\right)}^{i}\right)\cdot \delta \varepsilon_{x}^{i+1}$$

The functions *f*_1_ and *f*_2_ have different forms for the stress path under the yield surface and are different after the path has entered the yield surface. The forms of the functions *f*_1_ and *f*_2_ depend on the adopted constitutive model of the soil.

At $\delta \sigma_{22}^{i+1}=0,$ in effect of the successive increment of the horizontal strain $\delta \varepsilon_{x}^{i+1}$, the changes in stress state components and the increments of vertical strain will be as follows, respectively:

— for the path under the yield surface (regardless of the development degree of the constitutive model):
  (9) $$\delta \sigma_{11}^{i+1}=\frac{{\left(K_{et}+4\cdot G_{et}/3\right)}^{2}-{\left(K_{et}-2\cdot G_{et}/3\right)}^{2}}{\left(K_{et}+4\cdot G_{et}/3\right)}\cdot \delta \varepsilon_{x}^{i+1},\;\delta \sigma_{22}^{i+1}=0,\;\delta \sigma_{12}^{i+1}=0$$
  (10) $$\delta \sigma_{33}^{i+1}=\frac{2\cdot G_{et}\cdot \left(K_{et}-2\cdot G_{et}/3\right)}{\left(K_{et}+4\cdot G_{et}/3\right)}\cdot \delta \varepsilon_{x}^{i+1}\;\mathrm{and}\;\delta \varepsilon_{22}^{i+1}=-\frac{K_{et}-2\cdot G_{et}/3}{K_{et}+4\cdot G_{et}/3}\cdot \delta \varepsilon_{x}^{i+1}$$
— for the paths on the yield surface:
  (11) $$\delta \sigma_{11}^{i+1}=[\frac{4G_{et}^{2}\cdot H-3K_{et}\cdot {\left(H_{11}-H_{22}\right)}^{2}}{4G_{et}\cdot H-3H_{22}^{2}+3H\cdot K_{et}}+\frac{4G_{et}\cdot \left(3H\cdot K_{et}-H_{11}^{2}-H_{11}\cdot H_{22}-H_{22}^{2}\right)}{4G_{et}\cdot H-3H_{22}^{2}+3H\cdot K_{et}}]\cdot \delta \varepsilon_{x}^{i+1}$$
  (12) $$\delta \sigma_{22}^{i+1}=0,\;\delta \sigma_{12}^{i+1}=H_{12}\cdot \frac{3K_{et}\cdot \left(H_{22}-H_{11}\right)-2G_{et}\cdot \left(2\cdot H_{11}+H_{22}\right)}{4G_{et}\cdot H-3H_{22}^{2}+3H\cdot K_{et}}\cdot \delta \varepsilon_{x}^{i+1}$$
  (13) $$\begin{array}{l} \delta \sigma_{33}^{i+1}=[\frac{3K_{et}\cdot \left(H_{11}-H_{22}\right)\cdot \left(H_{22}-H_{33}\right)-4G_{et}^{2}\cdot H}{4G_{et}\cdot H-3H_{22}^{2}+3H\cdot K_{et}}\\ \;\;\;\;+\frac{2G_{et}\cdot \left(H_{22}^{2}-H_{22}\cdot H_{33}-H_{11}\left(H_{22}+2H_{33}\right)+3H\cdot K_{et}\right)}{4G_{et}\cdot H-3H_{22}^{2}+3H\cdot K_{et}}]\cdot \delta \varepsilon_{x}^{i+1} \end{array}$$
  (14) $$\delta \varepsilon_{22}^{i+1}=\frac{2\cdot G_{et}\cdot H+3\cdot H_{11}\cdot H_{22}-3\cdot H\cdot K_{et}}{3\cdot H\cdot K_{et}+4\cdot G_{et}\cdot H-3\cdot H_{22}^{2}}\cdot \delta \varepsilon_{x}^{i+1}$$

**Table 3:** Procedure for determining the stress state in the surface layer of the rock mass.

Step	Calculations
0	- definition of the stress state in situ according to Equation (6)
1	- assumption
	$\sigma^{i}=\sigma_{in\text{–}situ}$
	- determination of the size of the incremental step *δε_x_* and the auxiliary index *ε_b_^i^*:
	*ε_b_^i^* = 0
2	- determination of the increments of stress state components caused by the increment of horizontal strain *δε_x_* according to formulas (9)–(14).
3	- determination of the total components of stress state,
	$\sigma^{i+1}=\sigma^{i}+\delta \sigma^{i+1}$,
	determination of the auxiliary index
	*ε_b_*^*i*+1^ = *ε_b_^i^* + *δε_x_*
	and check if the condition *f* < 0 or *f* = 0 is fulfilled
4	- assumption
	$\sigma^{i}=\sigma^{i+1}$,
	and verification
	*ε_b_^i^* = *ε_b_*^*i*+1^ < *ε_x_* (15)
5	If the inequality (15) is satisfied, it is necessary to return to step 2; otherwise, the computation ends

#### 2.2.3. Limit States in the Subsoil Layer in the Modified Cam-Clay (MCC) Model

For the critical state model MCC, the plasticity function and the hardening law were presented in Equation (16). The functions that make up the plastic part of the matrix *D^ep^* in MCC are written in Equations (17) and (18). The derivation of these equations was described in detail by e.g., Springman et al. [55,56] and Zadroga [57]:

(16) $$f=q^{2}+M^{2}\cdot \left(p-p_{c}\right)\cdot p=0\;\mathrm{and}\;\delta p_{c}=p_{o}\cdot exp\left(\frac{\left(1+e\right)\cdot \varepsilon_{v}^{p}}{\lambda -\kappa }\right)\cdot \delta \varepsilon_{v}^{p}$$

(17) $$K_{f}=-exp\left(\frac{\left(1-e_{o}\right)\cdot \varepsilon_{v}^{p}}{\left(\lambda -\kappa \right)}\right)\cdot M\cdot p\cdot B,\;H_{ij}=\frac{1}{q}\cdot \left[3\cdot G_{et}\cdot s_{ij}+K_{et}\cdot q\cdot B\cdot \delta_{ij}\right],$$

(18) $$H=3\cdot G_{et}+K_{et}\cdot M\cdot B+K_{f}\;\mathrm{and}\;B=M\cdot \left(1+\ln\left(\frac{p}{p_{c}}\right)\right).$$

As a result of mining impact, the subsoil layer (not loaded with a structure) may be subjected to soil loosening, compaction and re-loosening processes. These phenomena are affected by the progress of the mining exploitation front. The effect of the location of face mining, speed exploitation was described in detail by other scientists, for example, Popiołek et al. [58] and Gogolewska and Jakubiak [59]. 

In critical state models, the soil loosening (compaction) state signals that the path enters the plasticity surface, belonging to the state boundary surface (SBS). As the soil loosens, the path moves along the SBS surface towards the critical state line (CSL), along with the change of the porosity index measurable in the unit volume of the soil.

**Figure 7:**
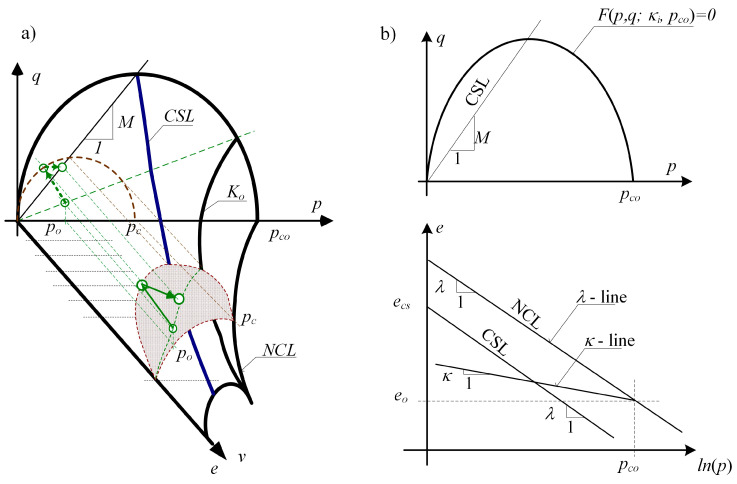
(**a**) State boundary surface, (**b**) identification of the parameters of the MCC model: *p*, *q*—stress invariants (upper Figure), *e*—porosity index, *p_co_*—over consolidation pressure (lower Figure).

Figure 7a presents the state boundary surface (SBS) characteristic for the critical state models (state surface), closing underneath all states possible in the soil. Figure 7b enables the identification of the parameters of the MCC model. The assumed parameters of the cohesive, overconsolidated soil were presented in Table 2; for the ratio *OCR* = 1 + q*/(*γ*⋅*z*). The critical state line CSL in the system of invariants (*p*, *q*) is shown in Figure 7. Moreover, porosity index *e* (changing along with the depth with the average stress (*p*) and average over consolidation pressure *p_c_*) was assumed according to Equation (19).

(19) $$e=e_{cs}-\left(\lambda -\kappa \right)\ln\left(p_{c}/2\right)-\kappa \ln\left(p\right)$$

In this study for the over-consolidated soil was used the Wroth formula (Equation (20) which was described in detail in [18,40]:

(20) $$K_{o}^{\left(OC\right)}=OCR\cdot K_{o}^{\left(NC\right)}-\frac{\nu }{1-\nu }\cdot \left(OCR-1\right)$$

Thus, the stresses in the in situ state (dependent on the consolidation state of the soil) can be written in Equation (21).

*σ*_11_ = *K_o_*·*σ*_22_, *σ*_22_ = *γ*·*z*, *σ*_33_ = *K_o_^O^σ*_22_, *σ*_12_ = 0 (21)

## 3. Results and Discussion

### 3.1. Model (C-M) in the Numerical Assessment of the Limit State Caused by Soil Loosening

This section presents the results for the case I, in which the subsoil was described by an elastic-ideally plastic model with the Coulomb-Mohr yield surface.

**Figure 8:**
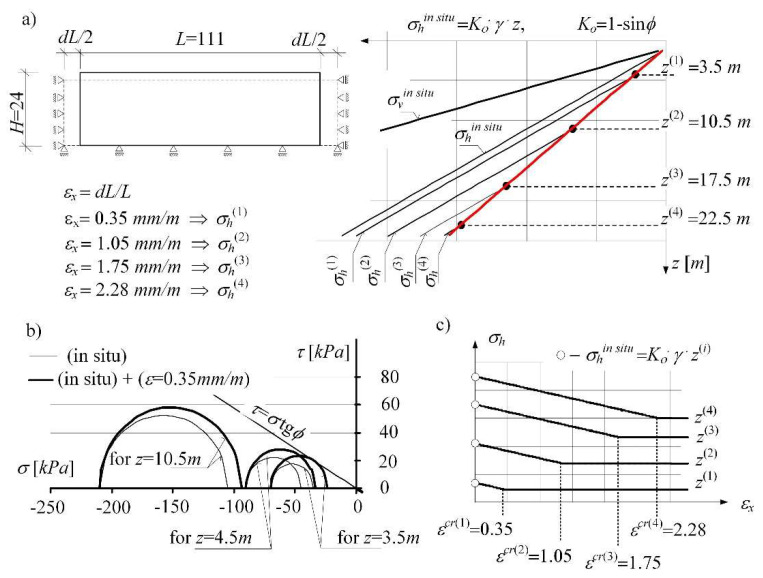
(**a**) Changes in the in situ state of stress of the horizontally loosened subsoil layer in the (C-M) model, (**b**) progressive damage in the soil, (**c**) determination of critical strains for different depths *z*.

With the increase in the incrementally enforced loosening of the soil (i.e., with the increase in the total strain of the soil *ε_x_*) the enlarging process of the zone is proceeding, in which the plastic equilibrium state preceding the plastic damage is fixed (Figure 8). In this zone, the horizontal stresses are gradually determined according to Equation (22). The results are marked with a red line in Figure 8a:

(22) $$\sigma_{h}\left(z\right)=K_{a}^{n}\cdot \sigma_{v}\left(z\right)=K_{a}^{n}\cdot \gamma \cdot z.$$

The value of the active pressure coefficient corresponding to Rankin’s state, in line with the theoretical solution, is: *K_a_* = (1 − *sinϕ*)/(1 + *sinϕ*). The value of the coefficient *K_a_^n^* determined numerically (for the angle of internal friction ϕ = 30°) is: $K_{a}^{n}$ = *K_a_* = 0.333 (Figure 8a).

### 3.2. The MCC Model in the Numerical Assessment of the Limit State Affected by Soil Loosening

**Figure 9:**
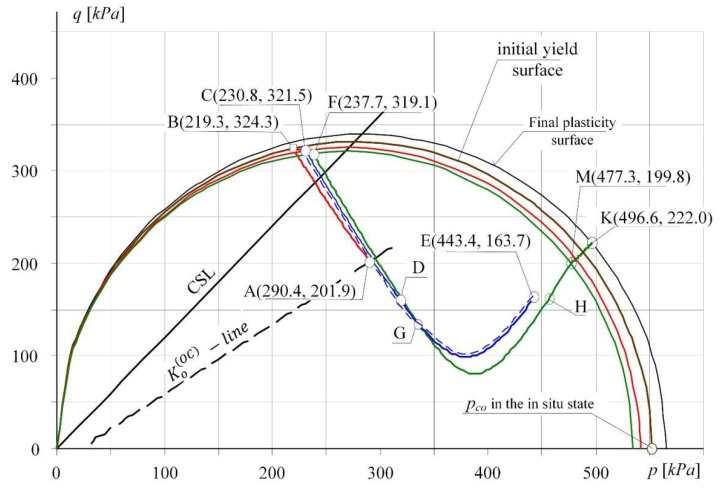
The course of stress path on the plane of invariants (*p*, *q*) for a specified point of the layer.

**Figure 10:**
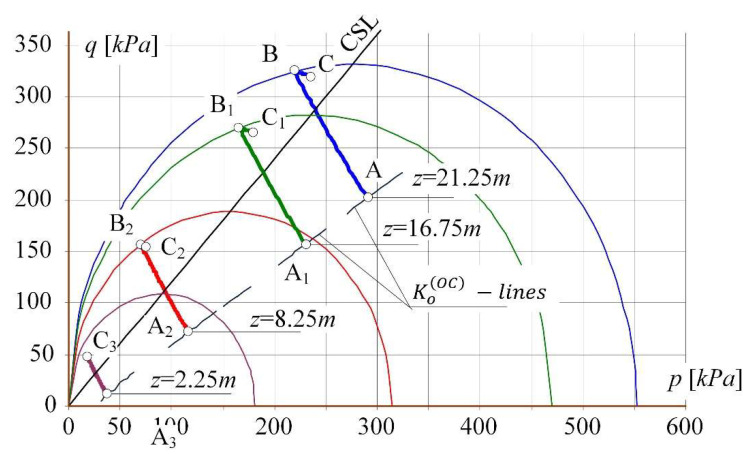
The stress paths in the system of invariants (*p*, *q*) for points located at different depths of the subsoil layer.

The analysis of the stress path course (Figure 9) was carried out for point A in Figure 9 and Figure 10 according to the procedure presented in Table 3. The path A-B-C in Figure 9 shows the behaviour of the subsoil layer (with assumed parameters, see Table 2) subjected to horizontal soil loosening to the strain *ε_x_* = 3.5 mm/m. While the path C-D-E shows the soil compacted to the initial state of *ε_x_* = 0. Subsequently, the soil was loosened again to the strain *ε_x_* = 5.25 mm/m (path E-D-C-F) and compacted again to its initial state of *ε_x_* = 0 mm/m (path F-G-H-M-K). It can be observed a transition from the state of over-consolidation of soil (point A beneath the SBS surface) to the state of normal consolidation—i.e., the entry of the path in the point B (at *ε_x_* = 1.53 mm/m) on the plasticity surface and at the same time on the state surface SBS. The compaction is accompanied by a change in soil state again, and the path descends (C-D-E) on the elastic wall beneath the state surface SBS. 

Figure 10 shows in the system of invariants (*p*, *q*) the course of stress paths for soil subjected to horizontal loosening to the strain *ε_x_* = 3.5 mm/m for four points located at different depth of the subsoil layer. Points A, A_1_, A_2_ and A_3_ are the starting points of the paths. The points: B (for *ε_x_* = 1.53 mm/m), B_1_ (for *ε_x_* = 1.75 mm/m), B_2_ (for *ε_x_* = 2.73 mm/m) defines the entry of the paths on the plasticity surfaces (about the values *p_co_* appropriate for the given depths *z*). The final position of the paths (for the horizontal loosening of *ε_x_* = 3.5 mm/m) is demonstrated by points C, C_1_, C_2_, and C_3_.

The assessment of soil behaviour needs to emphasise that the soil at a depth of *z* = 2.25 m was still in the over-consolidation state. The stress path A3—C3 did not achieve the plasticity surface (as opposed to the deeper points).

**Figure 11:**
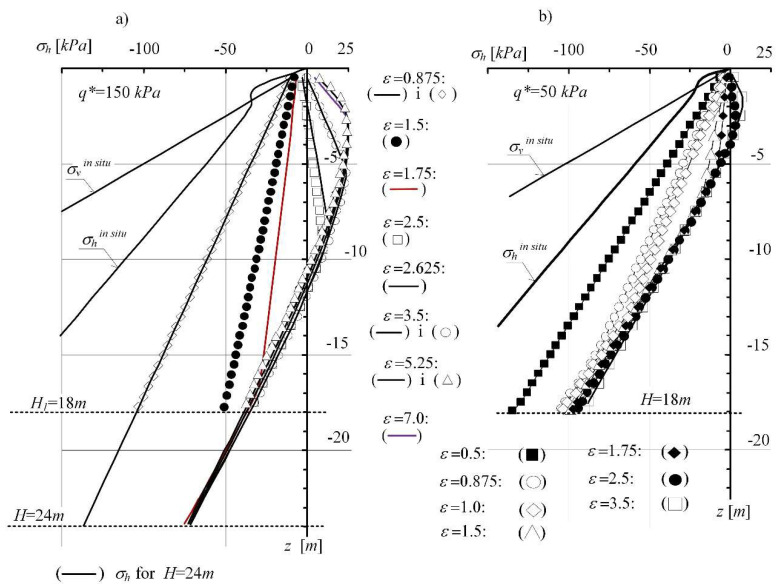
Formation of the state of limit equilibrium in the MCC subsoil model: (**a**) with q* = 150 kPa, (**b**) with q* = 50 kPa.

In critical state models, soil strength assessment in stress is coupled with the identification of the changes in volumetric strains in the space (*p*, *q*, *V*) or (*p*, *q*, *e*), where *p*, *q*—are the invariants of the stress state, *V*, *e*—specific volume and porosity index, respectively. In the next step, the development process of limit state in a loosening mining subsoil that has been subjected to effective stress in its past stress history larger than that existing at the present time was analysed (Figure 11). For the over-consolidated soil (of two different overburden values q*), the in situ stress distribution *σ_h_* according to Wroth was introduced. The subsoil (P_g_) defined by the overburden value: q* = 150 kPa (Figure 11a) and q* = 50 kPa (Figure 11b) was subjected to the increasing horizontal deformations, which resulted in its loosening.

Figure 11 presents the reduction of horizontal stress *σ_h_* progressing with the increase in soil loosening. The changing stresses (*σ_v_*, *σ_h_*’) form a path entering the SBS surface (representing the state of normal soil consolidation). With the progressing loosening of the subsoil, the stresses achieve the active limit state—the state of plastic equilibrium represented by the values (*σ_v_*, *K_a_*⋅*σ_v_*). The nature of the observed process is stable and independent of the consolidation rate and the assumed thickness of the layer (Figure 11a,b).

### 3.3. Numerical Simulations of the Active Limit State in the Layer Described by the Models (C-M) and MCC

**Figure 12:**
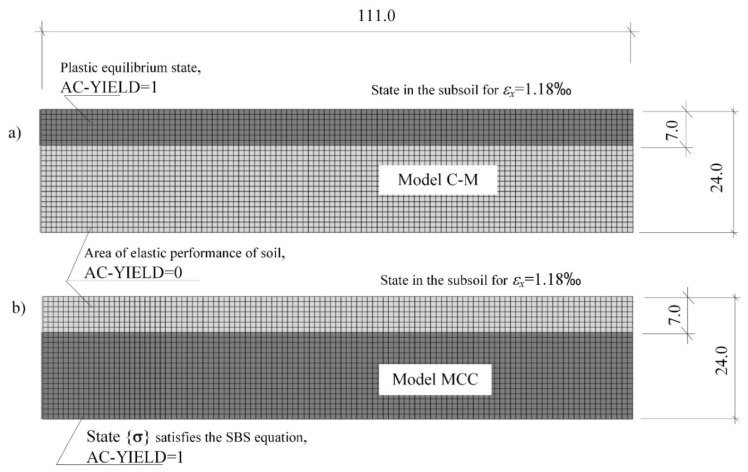
Forecasts of limit states in the subsoil subjected to loosening in the description with the constitutive compounds of: (**a**) Coulomb-Mohr, (**b**) Modified Cam-Clay critical state model.

Figure 12 presents the results of the numerical analysis of the subsoil layer described by the models (C-M) and MCC. By incrementally introducing the loosening of soil (reaching the value of $\varepsilon_{x}$ = 1.18‰), two different soil responses (depending on the constitutive model) to the load were obtained. In case I, when the mining area (P_g_) was described with the (C-M) model, the process of development and extension of the plasticity equilibrium zone occurs in the direction from the surface into the subsoil (Figure 12a). Moreover, using the (C-M) model, it can be observed:

— a simplified image of the non-linear behaviour of the loosened (compacted) soil,
— unrealistic values of critical strains accompanying the yielding.

While in the MCC model, the limit state of plasticity equilibrium is formed on the lower border of the subsoil area, and it expands with the increase in strains *ε_x_* towards the surface (Figure 12b). In addition, the results of the numerical simulation of subsoil loosening with the use of the MCC model were found to be reliable behaviour of mining subsoil, corresponding to field observations [35,36] and the results of laboratory tests [33,34,53].

### 3.4. The MCC Model in the Numerical Assessment of the Safety State of a Linear Structure

Based on the above, this section presents the applicability potential of the numerical analysis of the loosened subsoil layer for the assessment of protection elements (e.g., geo-matresses) used under linear structures in the areas subjected to mining and post-mining impacts. For this purpose, the numerical analysis was carried out to assess the horizontal strain of the subsoil loosening *ε_x_^o^*, above which the safety (stability) of the structure-subsoil system may be at risk. The thickness of the layer at risk was defined as critical and marked as *h^cr^*. Estimating (*ε_x_^o^* and *h^cr^*) can be useful for designing the protection of the structure-mining subsoil system (particularly roads pavement-subsoil system).

**Figure 13:**
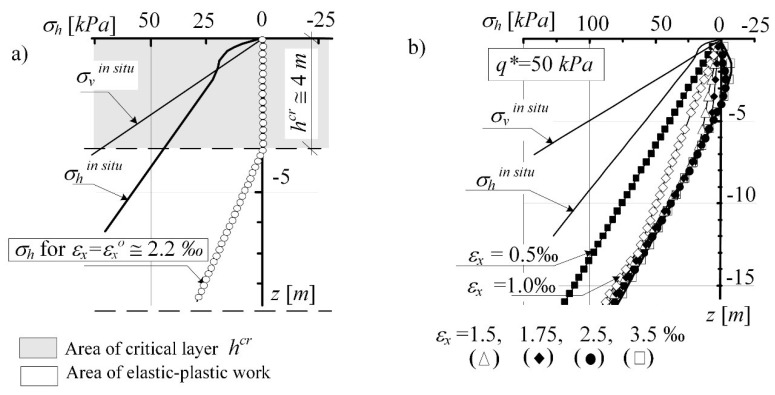
The critical layer *h^cr^* in the over-consolidated subsoil determined for *e_x_^o^* (**a**) the critical layer *h^cr^* in the over-consolidated subsoil determined for *ε_x_^o^*; (**b**) change of stress *σ_h_* as a result of increasing strain *e_x_^o^*.

**Figure 14:**
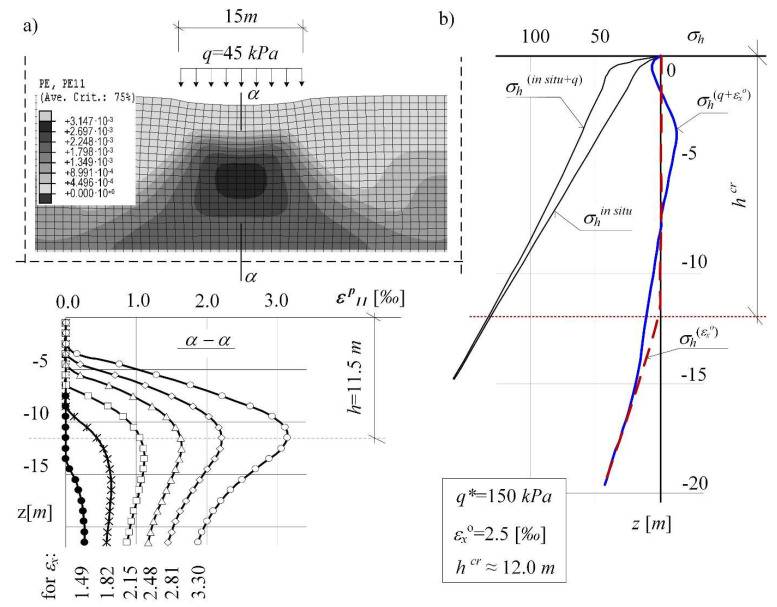
Numerical simulation of the “mechanism” of stability hazard for the structure-subsoil system. (**a**) numerical simulation of the “mechanism” of stability hazard for the structure-subsoil system; (**b**) stress state *σ_h_* for *ε_x_^o^*.

Figure 13 presents the results of analysis for over-consolidated soil with q* = 50 kPa. The results for strongly over-consolidated soil with q* = 150 kPa is presented in Figure 14. It presents a proposal for the simulation of the threat mechanism to the stability of the system structure-subsoil. The load is transferred from the road structure to the subsoil subjected to mining impacts. The “nucleus” of plastic strains, being formed under the loaded area, is increasing (expanding upwards) with the increase in the loosening of the subsoil, represented by a horizontal strain of the subsoil *ε_x_*. This results in a disturbance of the surface zone with *h^cr^* depth (determined in the subsoil, which is not loaded with a structure), see Figure 14b. In order to eliminate the stresses *σ_h_* > 0, lowering the permissible values of critical strain *ε_x_^o^* (of which exceeding this may result in the changes of soil state) was considered.

The investigation of areas subject to large displacement (obtained by forecasting, in-situ observed—Figure 1, and analysing) point out the risk of discontinuous surface ground deformations with comparatively large values of horizontal strains *ε_x_*. By tracking the changing function of horizontal stresses (Figure 13), it can be observed the identify the critical state of strain at which the horizontal stresses along the subsoil “strip” near the surface reach the final allowable value *σ_h_*’ = 0.

Estimating the values (*ε_x_^o^* and *h_kr_*) can be useful when assessing the optimal depth of the safety system elements’ location under the protected area, capable of reducing the strains transferred from the subsoil to a structure.

## 4. Summary and Conclusions

The study aimed to create a simple numerical model to assess the real state of subsoil being the building subbase in the mining or post-mining areas. This study indicated the important role of the history of stresses in subsoil model description on the real behaviour of the soil subjected to mining or post-mining deformations. Thus, it was determined that the proper constitutive description of the subsoil undergoing the loosening (due to mining or post-mining deformations) is indispensable to make the description of limit states formation in the soil more real. Moreover, the following detailed conclusions can be drawn on the basis of this experimental study’s results:

(1) The simulations involving the behaviour of the deforming subsoil layer using the Coulomb-Mohr model leads to the numerical restoration of the limit equilibrium state, consistent with the analytical solution. Still, it does not provide a proper interpretation of the processes occurring in the subsoil with the actual historical load record.
(2) The analyses based on the (C-M) model, suggesting the possibility of rapidly progressing damage resulting from the impact of strains *ε_x_* “enforce” an overly conservative approach to the designing of protections located in the upper layers of the subsoil under a structure (for example, Figure 2c).
(3) Through the application of the numerical analysis, using the Modified Cam-Clay critical state model, a realistic picture of the behaviour of subsoil subjected to mining (or post-mining) deformations was obtained.
(4) The responses of the soil model in the form of critical strains of the values which in the in situ state can cause discontinuous deformations of the surface and damage (cracks) of the structure interacting with the soil (Section 3.4, Figure 13) were determined.

In addition, in this study, it was demonstrated how the use of the equations of the MCC critical state model (in tabular form, Section 2.2) allows in a simple, analytical way to assess the risk of limit state at a selected depth under the surface of a loosening subsoil layer. To make real (to the design level) the proposition of a simulation method of the threat mechanism to the stability of the system structure-subsoil (from Figure 14), it would require to extend the numerical studies. Conscious and creative use of critical state models in research and design practice requires, in the authors' opinion, a detailed analysis involving the impact of the porosity index on the response of the MCC model under kinematic load. This will be the subject of further research.

## Figures and Tables

**Table 1:** Parameters for C-M model.

Parameter	Value
General modulus of elasticity, *E* (MPa)	30
Cohesion, *c* (MPa)	0
Angle of friction, *ϕ* (°)	30
Poisson ratio, *ν* (-)	0.30
Density, *γ* (kN/m^3^)	20

**Table 2:** Parameters for MCC model according to [50].

Parameter	Value
Overburden pressure, q* (kPa)	50 and 150
Slope of over consolidation line, *κ* (-)	0.0074
Slope of NCL line, *λ* (-)	0.066
Void ratio critical value, *e_cs_* (-)	0.30
Slope of critical line, *M* (-)	1.788
Poisson ratio, *ν* (-)	1.2
Density, *γ* (kN/m^3^)	20
$\mathrm{Coefficient}\;\mathrm{of}\;\mathrm{earth}\;\mathrm{pressure}\;\mathrm{at}\;\mathrm{rest},\;K_{o}^{\left(NC\right)}$ (-)	0.5

## Data Availability

Not applicable.
